# Alternative to MSCR Test: A Novel Rheological Method for Evaluating Asphalt Mastic Performance at High Temperatures

**DOI:** 10.3390/ma18235435

**Published:** 2025-12-02

**Authors:** Stefan Trifunović, Johannes Büchner, Michael P. Wistuba

**Affiliations:** Braunschweig Pavement Engineering Center (ISBS), Technische Universität Braunschweig, 38106 Braunschweig, Germany

**Keywords:** asphalt mastic, Dynamic Shear Rheometer, Multiple Stress Creep and Recovery, Single Shear Creep test, rheology, filler

## Abstract

The high-temperature performance of asphalt mastic is a critical factor influencing the resistance of asphalt mixtures to permanent deformation. Despite the importance of this material phase, no standardized test exists for evaluating asphalt mastic behaviour at high temperatures. Therefore, researchers often use the Multiple Stress Creep Recovery Test (MSCRT), originally designed for asphalt binder, although its applicability to asphalt mastic is limited. This study proposes a novel rheological method referred to as Single Shear Creep Test (SSCT) as a more robust alternative for assessing the performance of asphalt mastic at high temperatures. The SSCT applies a constant shear stress over an extended period, allowing for the determination of the steady-state creep rate as a rheological performance indicator. A comprehensive experimental program involving 45 asphalt mastic variants, produced by using 11 asphalt binder types, 15 mineral fillers, and different filler-to-asphalt binder ratios. Each variant was tested using both MSCRT and SSCT in a Dynamic Shear Rheometer (DSR). The results demonstrated that SSCT provides more consistent and rheological meaningful differentiation between materials. The results show that asphalt binder type and the filler-to-bitumen (f/b) ratio strongly influence asphalt mastic behaviour at high temperature. Filler type has a limited influence, except for hydrated lime.

## 1. Introduction and Background

Asphalt pavements consist of coarse aggregates, fine mineral aggregate (filler), and asphalt binder. In Europe, filler is defined as the fine aggregate with particles smaller than 0.063 mm (0.075 mm in the United States). During production of asphalt mixtures, these filler particles combine with the asphalt binder to form asphalt mastic, which acts as the primary adhesive binder phase in the asphalt mixture.

As to hot-rolled asphalt mixtures, the binder content as well as its rheological performance drive the formation of the grain structure during asphalt compaction, which is mainly responsible for the resistance of the asphalt pavement to traffic loading. Later, in the finished asphalt surface layer, the pavement stiffness and resistance to permanent deformation suffers when the asphalt is exposed to strong sunlight and high temperatures, the asphalt mastic softens, and the pavement becomes susceptible to permanent deformation and the formation of ruts.

Hence, the rheological properties and performances of asphalt mastic influence the stability of the asphalt mixture and have a decisive influence on the overall performance of the asphalt pavement [1]. Recent studies based on extensive rheological testing have confirmed a strong correlation between asphalt mastic rheology and asphalt mixture performance [2,3,4]. Incorporating mineral filler greatly increases the stiffness of the asphalt binder (the mastic’s modulus can be several times higher than that of pure asphalt binder). Thus, the proper selection and proportioning of asphalt binder and filler must be carefully coordinated to enhance the asphalt mixture’s long-term performance [5].

Currently, there is no standardized test method to evaluate the asphalt mastic’s performance at high temperatures in a Dynamic Shear Rheometer (DSR). Because of that, researchers are trying to adopt the tests developed for asphalt binder to evaluate asphalt mastic [6,7]. In particular, the Multiple Stress Creep and Recovery test (MSCRT), according to EN 16659 [8] or AASHTO TP 70-13 [9], was originally developed for characterizing polymer-modified asphalt binder. MSCRT has been frequently applied to asphalt mastic despite the lack of tailored protocols for asphalt mastic. Although MSCRT has been frequently applied to asphalt mastic, several limitations arise when the method is used beyond its original purpose. Asphalt mastic exhibits a substantially slower viscoelastic response than bitumen due to its high filler content, meaning that the standard MSCRT loading sequence of 1 s creep followed by 9 s recovery is typically too short for the material to reach steady-state flow or to complete its recoverable deformation [10,11,12,13]. As a result, the MSCRT parameters (non-recoverable creep compliance (Jnr) and percent recovery (R)) become dependent on the test duration rather than the essential material behaviour and therefore remain largely empirical and not physically meaningful for asphalt mastic [5,14]. To address these shortcomings, researchers have proposed creep–recovery tests with extended loading and recovery times. Santagata et al. [10] demonstrated that extended creep durations more reliably capture the steady-state deformation of asphalt mastic and allow clearer distinction between recoverable and non-recoverable strain components. Building on this concept, the present study introduces the Single Shear Creep Test (SSCT), which applies sufficiently long loading intervals to allow asphalt mastic to fully develop its time-dependent deformation behaviour, thereby providing a more robust method for high-temperature mastic evaluation. The SSCT is also included in Annex E of the German technical guideline for testing asphalt mastic in DSR, where it is proposed as a recommended method for evaluating the high-temperature performance of asphalt mastic [15].

## 2. Objective and Methodology

This study investigates how the high-temperature performance of asphalt mastic can be accurately assessed using an alternative rheological test method in DSR. The focus is on evaluating the SSCT as a potential alternative to the commonly used MSCRT. The MSCRT is known to have limitations such as the inability to fully capture steady-state deformation behaviour [10,11,12]. For this reason, the SSCT is explored as a more stable and reliable approach for evaluating asphalt mastic performance at high temperature.

In addition, this study aims to analyse the influence of asphalt binder type, filler type, and f/b ratio on the rheological behaviour of asphalt mastic at high temperature. A total of 45 different asphalt mastic variants are prepared for testing by SSCT and MSCRT. These asphalt mastic variants are prepared by using 11 different asphalt binder types, including unmodified, polymer-modified, and long-term aged asphalt binder, in combination with 15 different mineral fillers. The binder types were selected to represent the most commonly used modified and unmodified asphalt binders in the asphalt construction sector. The selected fillers cover a wide range of chemical compositions, particularly with respect to silicon dioxide (SiO_2_) content, which is known to affect the reactivity and interfacial bonding between filler and asphalt binder [16].

The f/b ratios used in this study range from low to moderately high values, representing typical proportions used in practical asphalt mixture design. This allows for a detailed evaluation of how increasing filler content affects deformation resistance at high temperature.

A final objective of this study is to compare these two test methods, not only in terms of their results but also in their application across two different DSR systems from manufacturers A and B. Since the construction principle of a rheometer can influence the results of asphalt binder and mastic testing, this comparison aims to assess the consistency and robustness of both methods under varying instrumentation conditions [17,18].

**Figure 1:**
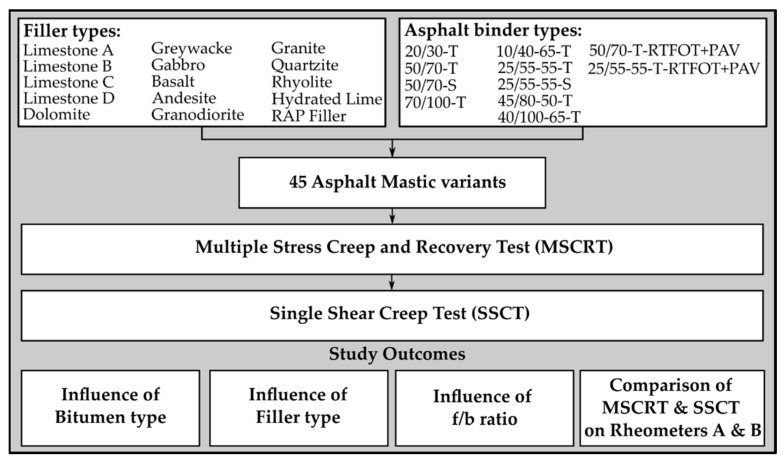
Flowchart of the methodology.

Figure 1 shows the methodology of this study, including the materials used, test methods, and outcomes.

## 3. Materials

### 3.1. Asphalt Binder

In this study, only commercially available standard asphalt binders are used. A total of nine asphalt binder types commonly available in the German market are selected. The unmodified asphalt binders include soft penetration grade 70/100, medium penetration grade 50/70, and hard penetration grade 20/30. The polymer-modified asphalt binders (PmBs) used are 10/40-65, 25/55-55, 45/80-50, and 40/100-65.

**Figure 2:**
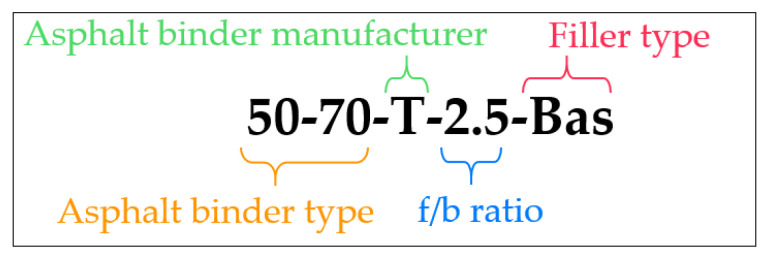
Example for labelling the asphalt mastic variants: asphalt binder type, asphalt binder manufacturer, f/b ratio, and filler type.

Penetration grade asphalt binder 50/70 and PmB 25/55-55 are among the most frequently applied asphalt binder in German road construction, and because of that, this study includes samples of these two asphalt binders from two different manufacturers (labelled as T and S, see Figure 2). Furthermore, long-term aged versions of these two asphalt binder types are included as well. Aging was performed in two stages, short-term aging using the Rolling Thin Film Oven Test (RTFOT) in accordance with EN 12607-1 [19], followed by long-term aging for 20 h using the Pressure Aging Vessel (PAV) in accordance with EN 14769 [20]. The asphalt binders were aged in the absence of filler to eliminate any potential influence of the filler on the aging process. Accordingly, some asphalt mastic variants are labelled with the code “RTFOT+PAV” to indicate the use of aged asphalt binder.

### 3.2. Filler

In this study, filler is defined as the mineral fraction of aggregate with a particle size smaller than 0.063 mm. A total of 15 different filler types were investigated, selected to represent the most commonly used fillers in road construction as well as to provide a wide range of fillers with a broad spectrum of silicon dioxide (SiO_2_) content. This was based on the assumption that the SiO_2_ content significantly influences the physicochemical reactivity between filler and asphalt binder. Among the fillers in this study, there is also an artificial filler, hydrated lime, which is occasionally used in small proportions in asphalt mixtures. Its pronounced stiffening effect arises from the reactive hydroxyl groups on the lime surface, which interact strongly with the polar components of the asphalt binder and therefore cannot be directly linked to its SiO_2_ content. Additionally, one of the fillers is obtained from Reclaimed Asphalt Pavement (RAP) through a hot extraction process according to EN 12697 [21].

The physical and chemical properties of the fillers were characterized through a series of standardized tests (Table 1), including the following:

- Particle density determination according to EN 1097-7 [22];
- Measurement of air void content using the Rigden method, in accordance with EN 1097-4 [23];
- Delta ring and ball softening point test following EN 13179-1 [24];
- Chemical composition analysis via X-ray fluorescence (XRF) according to ISO 29581-2 [25];
- Evaluation of specific surface area using the BET method in accordance with DIN ISO 9277 [26].

### 3.3. Asphalt Mastic

To ensure the production of homogeneous asphalt mastic samples, the filler is first sieved through a 0.063 mm sieve and then dried to a constant mass. It is subsequently preheated for at least two hours at the designated mixing temperature, while the asphalt binder is heated to the same temperature only immediately prior to mixing. The mixing is conducted at 160 °C for asphalt mastic variants containing unmodified asphalt binder and at 180 °C for those with polymer-modified asphalt binder. The preheated filler is gradually added to the asphalt binder using a dosing spoon and simultaneously mixed in asphalt binder. Once the entire amount of filler is added to the asphalt binder, the asphalt mastic is stirred for a minimum of two minutes and a maximum of five minutes, as recommended by Wistuba et al. [18].

A total of 45 asphalt mastic variants were prepared to systematically evaluate the influence of each mastic component. To assess the influence of the asphalt binder type, all 11 asphalt binder types were combined with the same filler, the most commonly used limestone filler (Limestone A). These asphalt mastic variants were prepared with a f/b ratio of 1.0, which represents a realistic proportion typically found in asphalt mixtures. Similarly, to examine the influence of the filler type, asphalt mastic variants were produced using 15 different fillers, while keeping the asphalt binder (50/70 T) and the f/b ratio (1.0) constant. In typical asphalt concrete (AC) mixtures, the f/b ratio generally ranges between 0.5 and 2.5. The optimum f/b ratio is crucial, as it determines the thickness of the binder film coating the aggregate particles and thus directly influences the workability, stability, and long-term durability of the asphalt mixture. For this reason, the present study investigates a wide range of f/b ratios from 0.5 to 2.5 while keeping the same asphalt binder type (50/70 T) and filler (Limestone A).

The asphalt mastic variants are systematically labelled using a four-part label scheme (see Figure 2). The first component of the label identifies the type of asphalt binder used, which may include asphalt binder types mentioned in Section 3.1. The second component specifies the asphalt binder manufacturer, with two manufactures labelled as “S” and “T”. The third component of the label refers to the filler-to-bitumen (f/b) mass ratio, indicating the proportion of filler mass relative to the mass of bitumen. A mass-based definition is used because mass dosing is standard in asphalt mixture production and laboratory mastic preparation, making it the most practical and commonly applied approach. The fourth and final component indicates the type of filler used, based on the abbreviations provided in Table 1.

## 4. Test Methods

The asphalt mastic variants are tested using MSCRT and SSCT test methods in the DSR. This study also includes a comparative evaluation of these two test methods performed with DSR instruments from manufacturers A and B. The results presented in Section 5.1, Section 5.2 and Section 5.3 are obtained using the DSR device supplied by manufacturer B.

### 4.1. Single Shear Creep Test (SSCT)

The SSCT for asphalt mastic evaluation was developed at Braunschweig Pavement Engineering Centre at Technical University of Braunschweig, initially as part of the doctoral research of Johannes Büchner [3,13], and later further advanced within the framework of the research project “RHEMAS” [18]. SSCT is performed in a DSR using a parallel plate geometry with a diameter of 25 mm and a gap of 1 mm between the plates. A constant shear stress of 0.5 kPa is applied for 60 min at a test temperature of 60 °C to measure the creep rate during the quasi-linear phase. These parameters were selected during the development of the SSCT to ensure that all asphalt mastic variants, regardless of their stiffness, could reach the quasi-linear phase. The original SSCT test method included a 60 min recovery phase, but this phase was excluded from the procedure, as the recovery parameter did not yield meaningful insights into asphalt mastic performance at high temperature [13]. The main result of the SSCT is the creep rate, expressed as the percentage increase in shear strain per second. A higher creep rate means that the material is more prone to permanent deformation. SSCTs were performed on a minimum of two specimens per asphalt mastic variant. If the deviation between the two results for creep rate exceeded 5%, an additional specimen was tested until two replicates met this criterion. Reported values represent the mean of the accepted specimens.

### 4.2. Multiple Stress Creep and Recovery Test (MSCRT)

The MSCRT is a standardized method for evaluating asphalt binder, defined in EN 16659 [8]. It is designed to assess the resistance of asphalt binder to permanent deformation under repeated loading conditions. Although originally developed for the asphalt binder, this test method is also commonly used to evaluate asphalt mastic, as it provides valuable information on the performance of asphalt mastic at high temperature.

The test is conducted using a DSR with a parallel plate geometry with a plate diameter of 25 mm and a gap of 1 mm. A series of ten cycles of creep and recovery are applied at a constant temperature between 50 and 70 °C. Each cycle consists of a 1 s creep phase followed by a 9 s recovery phase. The creep phase is conducted under a constant shear stress of 3.2 kPa or 0.1 kPa. The primary parameters obtained are the non-recoverable creep compliance (Jnr) and percent recovery (R), which reflect the material’s susceptibility to rutting and its elastic recovery potential. MSCRT were performed on a minimum of two specimens per asphalt mastic variant. If the deviation between the two results (either Jnr or R) exceeded 5%, an additional specimen was tested until two replicates met this criterion. The reported values represent the mean of the accepted specimens.

## 5. Results

This chapter presents a comprehensive evaluation of the high-temperature behaviour of asphalt mastic based on the results obtained from the SSCT and MSCRT test methods. The influence of asphalt binder type, filler type, and filler-to-binder ratio on creep rate, creep compliance, and percent recovery is examined in detail. The chapter also includes a comparative analysis of measurements performed on DSR devices from two different manufactures, as well as a Spearman correlation analysis used to investigate the relationship between filler properties and the rheological response of asphalt mastic. The combined findings provide a solid basis for evaluating the suitability of the SSCT as an alternative method to the MSCRT for assessing high-temperature asphalt mastic behaviour.

### 5.1. Influence of Asphalt Binder Type on High-Temperature Asphalt Mastic Performance

The influence of asphalt binder type on the high-temperature performance of asphalt mastic is evaluated based on the creep rate derived from the SSCT, as well as the percent recovery and creep compliance obtained from MSCRT.

**Figure 3:**
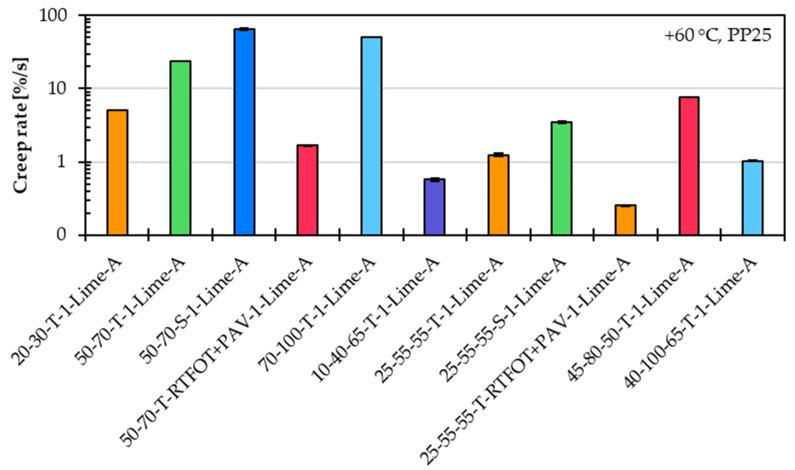
Creep rate of asphalt mastic variants prepared with limestone filler A, with an f/b ratio of 1, and eleven different asphalt binder types.

Figure 3 show the creep rate values in a logarithmic scale for eleven asphalt mastic variants prepared with limestone filler A and a constant f/b ratio of 1, and eleven different asphalt binder types, as described in Section 3.1.

The creep rate results clearly demonstrate that the type of asphalt binder has a significant influence on the high-temperature performance of the asphalt mastic. Notably, considerable differences in performance are also observed among asphalt mastic variants prepared with the same asphalt binder grade but sourced from different manufacturers. Furthermore, the incorporation of a modified asphalt binder lead to a substantial reduction in creep rate, indicating enhanced resistance to permanent deformation. Asphalt mastic variants containing a long-term aged asphalt binder showed clearly lower creep rates as well, reflecting increased stiffness and reduced susceptibility to creep under stress at high temperature.

**Figure 4:**
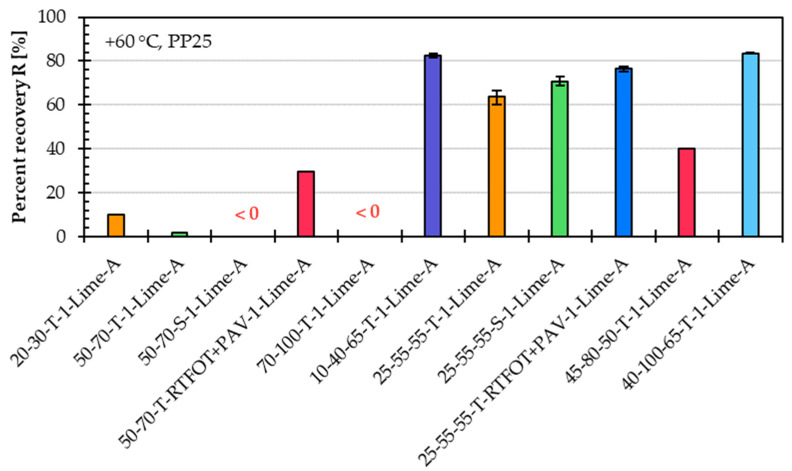
Percent recovery of asphalt mastic variants prepared with limestone filler A, an f/b ratio of 1, and eleven different asphalt binder types.

**Figure 5:**
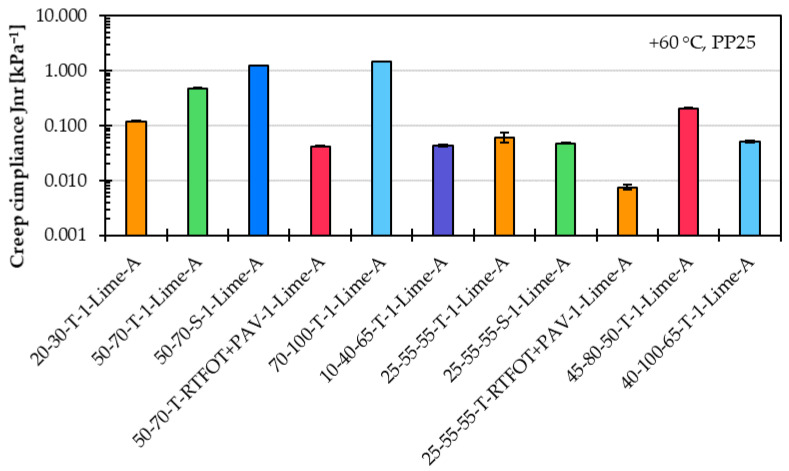
Creep compliance of asphalt mastic variants prepared with limestone filler A, an f/b ratio of 1, and eleven different asphalt binder types.

Figure 4 shows the percent recovery, and Figure 5 shows in logarithmic scale the creep compliance for the same eleven asphalt mastic variants prepared with different asphalt binder types.

Similar to the creep rate results, the percent recovery and creep compliance results further confirm that the asphalt binder type has a significant influence on the high-temperature performance of the asphalt mastic. The percent recovery values clearly distinguish between the mastic variants made with unmodified and modified asphalt binder types. Asphalt mastics with unmodified binders, such as 20/30-T-1-Lime-A and 50/70-T-1-Lime-A, exhibited low percent recovery values in the range of only a few percent. Even negative recovery is observed in variants containing softer unmodified binders, such as 50/70-S-1-Lime-A and 70/100-T-1-Lime-A, indicating poor elastic response. These findings further support the notion that the MSCR test is not well suited for evaluating a unmodified asphalt binder or asphalt mastic variants based on such an asphalt binder. In contrast, asphalt mastic variants incorporating long-term aged asphalt binder types (e.g., 50/70-T-RTFOT+PAV-1-Lime-A and 25/55-55-T-RTFOT+PAV-1-Lime-A) showed improved percent recovery, suggesting that oxidative aging increases the elastic response and contributes positively to recovery performance.

Creep compliance results similarly highlight the distinction between asphalt mastic variants prepared with unmodified and modified asphalt binder types. Mastic variants incorporating modified binders consistently exhibited lower creep compliance values, indicating improved resistance to permanent deformation. However, unlike the creep rate parameter, creep compliance did not reveal substantial differences between mastic variants made with asphalt binders of the same grade but sourced from different manufacturers. Additionally, asphalt mastic variants containing a long-term aged asphalt binder demonstrated reduced creep compliance, suggesting that increased binder stiffness due to aging has a positive effect on mastic performance at high temperature.

### 5.2. Influence of Filler Type on High-Temperature Asphalt Mastic Performance

The influence of filler type on the high-temperature performance of asphalt mastic is also evaluated as in the previous subchapter based on the creep rate derived from the SSCT and the percent recovery and creep compliance obtained from the MSCRT.

**Figure 6:**
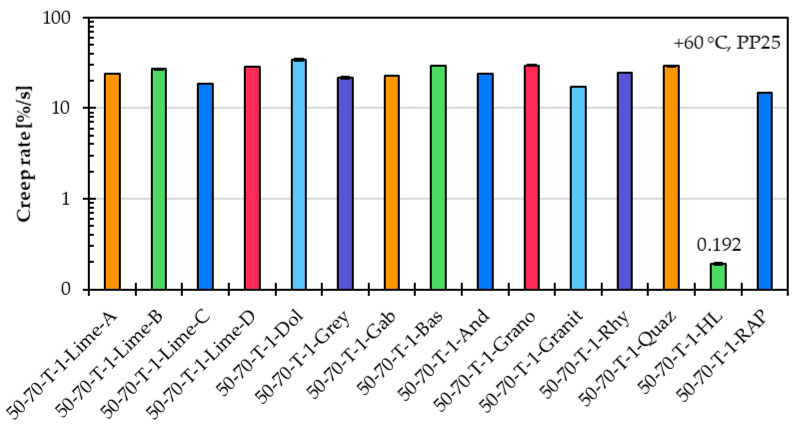
Creep rate of asphalt mastic variants prepared with unmodified asphalt binder 50/70 (T), an f/b ratio of 1, and fifteen different filler types.

Figure 6 shows in a logarithmic scale the creep rate values for fifteen asphalt mastic variants prepared with unmodified asphalt binder 50/70 (T), a constant f/b ratio of 1, and fifteen different filler types, as described in Section 3.2.

The creep rate results indicate that the influence of filler type on the high-temperature performance of asphalt mastic is relatively limited. However, a pronounced stiffening effect was observed in the case of hydrated lime, which resulted in a notably low creep rate of just 0.192%/s. No clear correlation was found between the physical and chemical properties of the fillers presented in Table 1 and the measured creep rates, except for the ∆Ring and Ball values. It was observed that fillers with higher ∆Ring and Ball values usually result in asphalt mastics with lower creep rates. This correlation is particularly evident in the cases of asphalt mastic variants prepared with Limestone C, Greywacke, Granite, RAP filler, and most notably hydrated lime, which serves as an extreme example of this effect.

**Figure 7:**
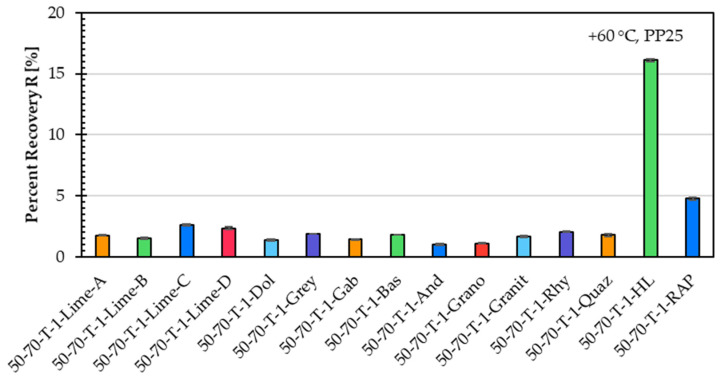
Percent recovery of asphalt mastic variants prepared with an unmodified asphalt binder 50/70 (T), a f/b ratio of 1, and fifteen different filler types.

**Figure 8:**
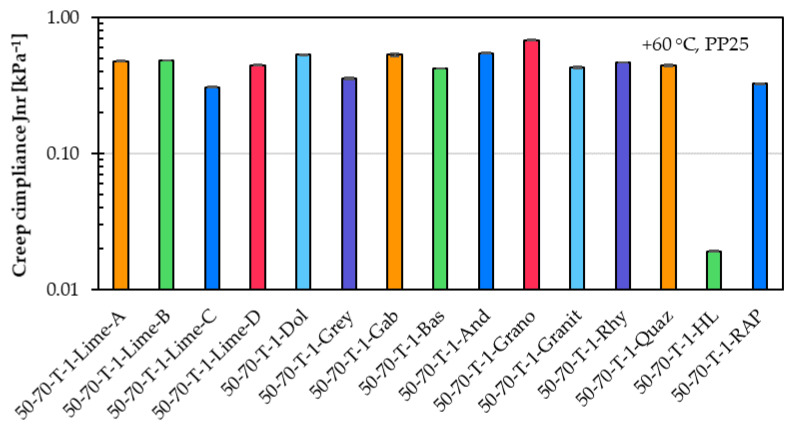
Creep compliance of asphalt mastic variants prepared with an unmodified asphalt binder 50/70 (T), a f/b ratio of 1, and fifteen different filler types.

Figure 7 shows the percent recovery, and Figure 8 shows in a logarithmic scale the creep compliance for the same fifteen asphalt mastic variants prepared with different filler types. Similar to the creep rate results, the percent recovery and creep compliance results confirm that the filler type has a limited influence on the high-temperature performance of the asphalt mastic.

Since all of these fifteen asphalt mastic variants are prepared with an unmodified asphalt binder, the percent recovery values are very low (around 2%) and offer limited insight into performance differences. However, due to the stiffening effect of hydrated lime and the smaller effect of the RAP filler, slightly higher percent recovery values were observed for the mastic variants incorporating these two filler types.

The creep compliance results closely correspond to the creep rate findings and confirm the same correlation. Filler types with higher ∆Ring and Ball values exhibit lower creep compliance, indicating better resistance to permanent deformation at high temperatures.

Table 2 shows the Spearman correlation coefficients between filler properties and the SSCT and MSCRT parameters. The analysis reveals several clear trends. Filler density shows a strong positive correlation with creep rate (ρ = 0.604) and creep compliance (ρ = 0.679), indicating that asphalt mastic variants with denser fillers show higher deformation under load. In contrast, ΔR&B shows a pronounced negative correlation with creep rate (ρ = −0.744), suggesting that fillers causing larger increases in softening point contribute to improved resistance to creep deformation. This clear correlation appears only for creep rate; the correlation between ΔR&B and creep compliance is noticeably weaker (ρ = −0.366). This may indicate that the relatively short creep period in MSCRT is insufficient for the mastic to express the full influence of the filler stiffening effect. Consequently, the stiffening trend measured by ΔR&B is only partially captured by creep compliance. Specific surface area demonstrates moderate negative correlations with both creep rate and creep compliance, consistent with the expected stiffening effect of finer fillers with higher surface area. The percent recovery shows moderate to strong correlations with several filler properties, such as the negative correlation with density (ρ = −0.686) and the positive correlation with specific surface area (ρ = 0.411), indicating that filler mineralogy influences the elastic response measured by MSCRT.

### 5.3. Influence of f/b Ratio on High-Temperature Asphalt Mastic Performance

The influence of the f/b ratio on the high-temperature performance of the asphalt mastic is evaluated, as in the previous two subchapters, based on the creep rate derived from the SSCT, as well as the percent recovery and creep compliance obtained from the MSCRT.

**Figure 9:**
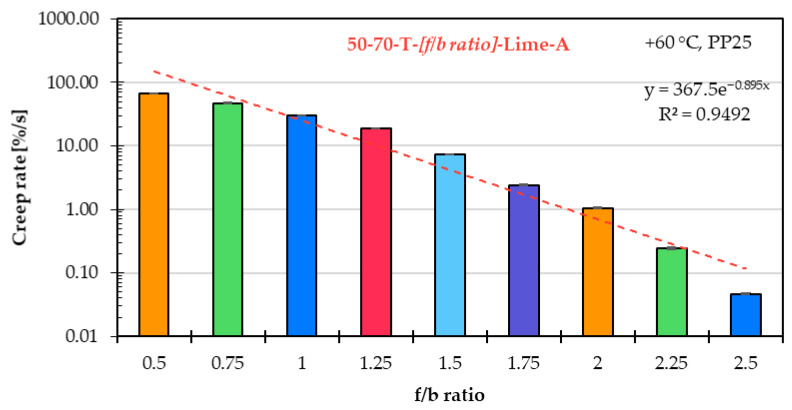
Creep rate of asphalt mastic variants prepared with unmodified asphalt binder 50/70 (T), limestone filler A, and f/b ratios ranging from 0.5 to 2.5.

Figure 9 shows the creep rates in the logarithmic scale of asphalt mastic variants prepared with unmodified asphalt binder 50/70 (T) and limestone filler A, covering f/b ratios from 0.5 to 2.5. The correlation between the f/b ratio and creep rate can be well approximated by a linear curve when the creep rate is plotted on a logarithmic scale. The marked decrease in creep rate illustrates the substantial stiffening effect associated with increasing the f/b ratio. Based on these results, it can be concluded that the f/b ratio has a strong influence on the high-temperature performance of asphalt mastic.

**Figure 10:**
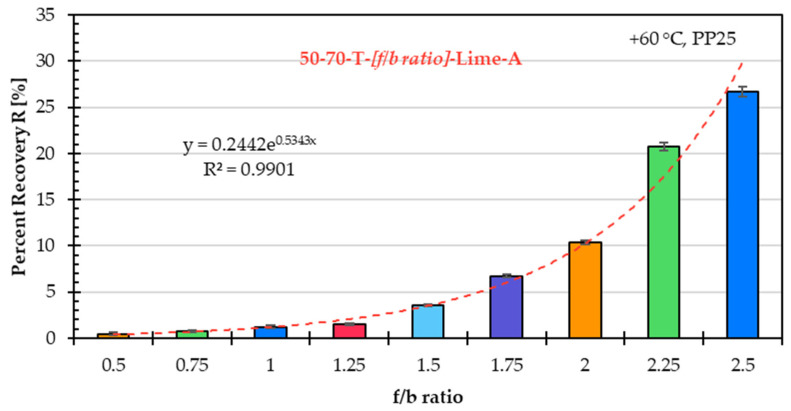
Percent recovery of asphalt mastic variants prepared with unmodified asphalt binder 50/70 (T), limestone filler A, and f/b ratios from 0.5 to 2.5.

**Figure 11:**
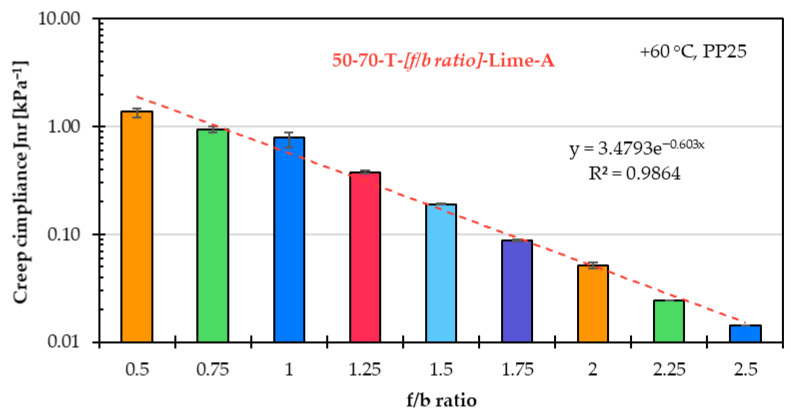
Creep compliance of asphalt mastic variants prepared with unmodified asphalt binder 50/70 (T), limestone filler A, and f/b ratios from 0.5 to 2.5.

Figure 10 shows the percent recovery, and Figure 11 shows in a logarithmic scale the creep compliance for the same asphalt mastic variants prepared with unmodified asphalt binder 50/70 (T), limestone filler A, and nine f/b ratios.

The correlation between percent recovery and the f/b ratio shows a clear logarithmic trend. An f/b ratio above 1.5 shows a strong positive influence on the percent recovery, indicating improved resistance to deformation at high temperatures; however, this comes at the cost of reduced low-temperature performance due to the increased stiffening effect.

The creep compliance shows the same linear correlation to the f/b ratio as creep rate does while it is plotted on a logarithmic scale.

Similar to the creep rate results, the percent recovery and creep compliance findings confirm that the f/b ratio has a significant influence on the high-temperature performance of asphalt mastic.

### 5.4. Comparative Analysis of MSCRT and SSCT Across Different DSR Device Manufacturers

The MSCRT and SSCT are conducted using two different DSRs (referred to as Rheometer A and Rheometer B), both located in the same laboratory and operated by the same technician. For reliability, each reported value is based on at least two measurements. If the deviation between the two results exceeded 5%, an additional specimen was tested until two replicates met this criterion. A total of 10 representative asphalt mastic variants are selected for the comparison, covering a wide range of material properties. These include variants with soft, medium, and stiff unmodified asphalt binders (20/30, 50/70, and 70/100), as well as aged asphalt binder (50/70 after RTFOT and PAV aging) and a polymer-modified binder (25/55-55). Additionally, the dataset includes asphalt mastic variants with various filler types (Gabbro, Andesite, and Granite) and with both low and f/b ratios (0.5 and 2.0), ensuring that the comparison accounts for diverse rheological behaviours.

**Figure 12:**
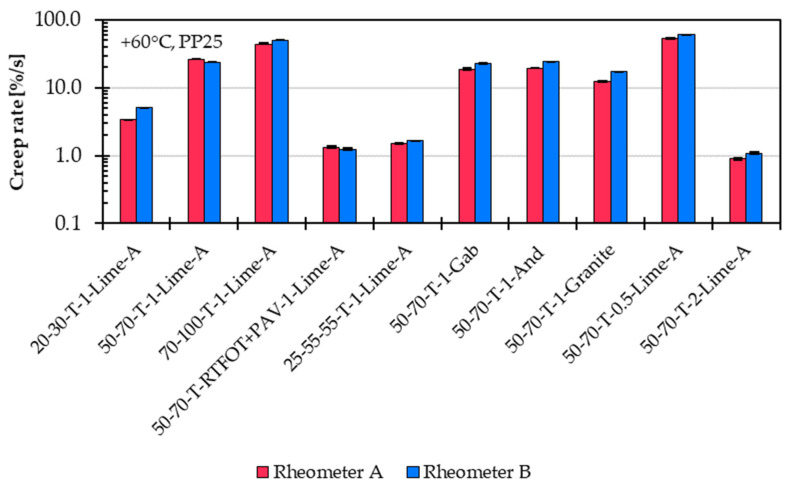
Comparison of creep rate results from the Single Shear Creep Test (SSCT) at 60 °C for 10 representative asphalt mastic variants measured using Rheometer A and B.

Figure 12 shows the comparison of creep rate results from the SSCT using Rheometers A and B. The measurements are quite consistent, with Rheometer B showing a variation of about 4% and Rheometer A about 8% from their average values. Overall, Rheometer B tends to give slightly higher creep rates. The biggest difference between the two devices is around 40% for the stiff asphalt mastic variant 20-30-T-1-Lime-A.

**Figure 13:**
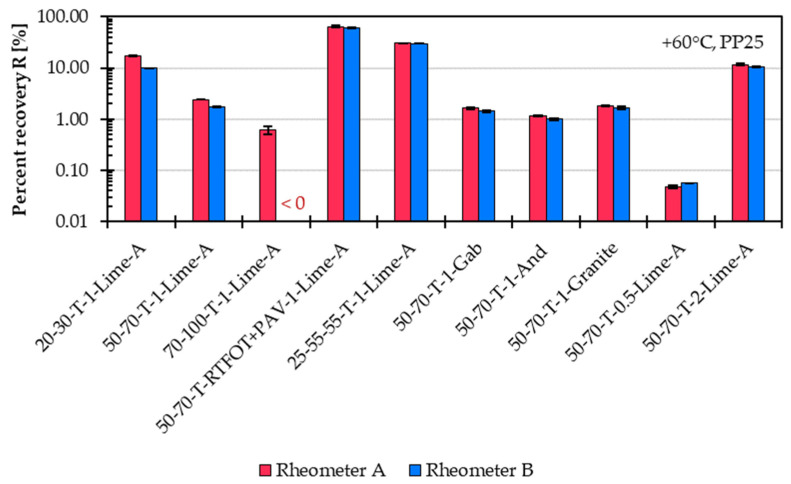
Comparison of percent recovery results from the Multiple Stress Creep and Recovery Test (MSCRT) at 60 °C for 10 representative asphalt mastic variants measured using Rheometer A and B.

**Figure 14:**
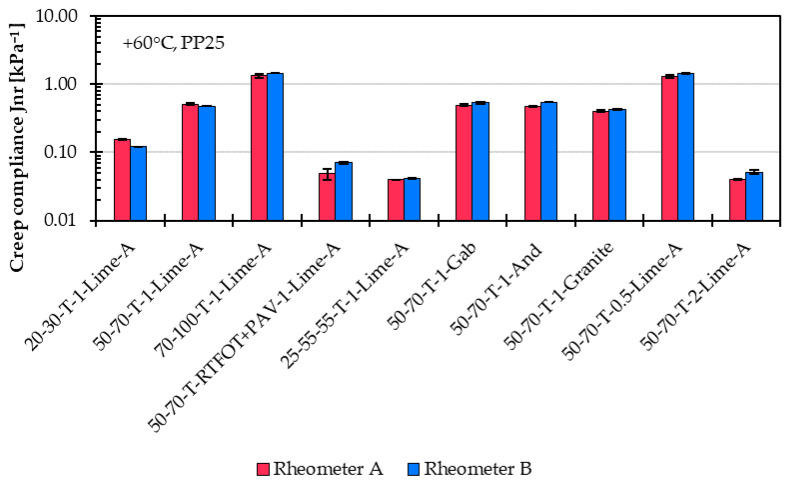
Comparison of creep compliance results from the Multiple Stress Creep and Recovery Test (MSCRT) at 60 °C for 10 representative asphalt mastic variants measured using Rheometer A and B.

The results obtained with both rheometers from the MSCRT are compared in Figure 13 for percent recovery and in Figure 14 for creep compliance.

The repeatability of the percent recovery is approximately 0.5% for Rheometer B and around 1.9% for Rheometer A. The lower repeatability observed for Rheometer A is mainly linked to measurement variations in the mastic variant 50-70-T-RTFOT-PAV-1-Lime-A. The percent recovery values measured with Rheometer A are up to 8% higher than those from Rheometer B. This difference remains within the comparison precision limit of 12 percent as defined by EN 16659 [8].

For creep compliance, the repeatability is about 8% for Rheometer B and approximately 13% for Rheometer A, based on their respective average values. These variations are a little bit higher than those observed in the creep rate results from the SSCT. The overall difference between the two devices is around 27%, which is within the acceptable comparison precision of 43% according to EN 16659 [8]. In general, Rheometer A tends to report slightly lower creep compliance values compared to Rheometer B.

**Figure 15:**
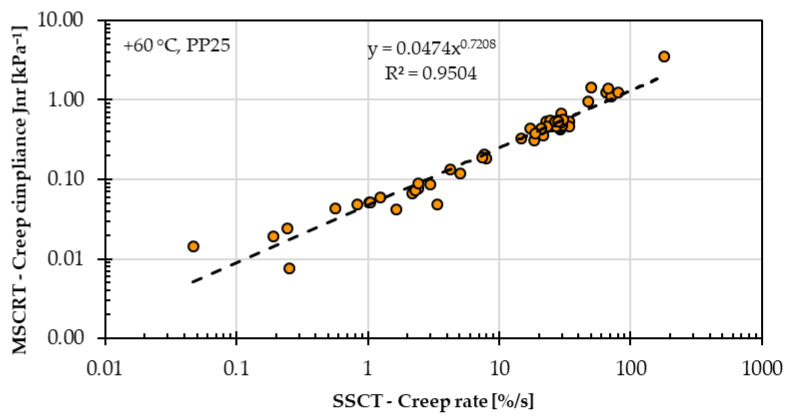
Comparison of creep compliance from the Multiple Stress Creep and Recovery Test (MSCRT) and creep rate from the Single Shear Creep Test (SSCT) at 60 °C for all 45 asphalt mastic variants investigated in this study.

Figure 15 shows the creep compliance values from the MSCRT and the creep rate values from the SSCT at 60 °C for all 45 asphalt mastic variants evaluated in this study. A clear linear correlation is observed between creep compliance and creep rate when both parameters are plotted on a logarithmic scale. A strong linear relationship was observed between these two parameters (R^2^ = 0.965, *p* < 0.001), confirming the statistical significance of the correlation. The repeatability of all SSCT and MSCRT results was evaluated according to ISO 5725-2 [27] using the 95% confidence level (2.77 × Sr). For all asphalt mastic variants, the repeatability values are 4.47% for creep rate (SSCT), 5.03% for creep compliance (MSCRT), and 6.77% for percentage recovery (MSCRT). All of these results confirm that both test methods provide comparable outcomes for assessing the high-temperature performance of asphalt mastic.

It is important to emphasize that MSCRT is an empirical test method originally developed for unmodified bitumen, whereas the SSCT captures a more physically meaningful representation of the material’s viscoelastic response. The SSCT applies a constant shear stress over an extended period, allowing the mastic to approach a quasi-linear steady-state regime in which the measured creep rate reflects the intrinsic rheological behaviour. In contrast, the short 1 s loading period in MSCRT often prevents highly filled systems from reaching steady deformation, which limits the interpretability of MSCRT parameters. Similar findings have been reported by Santagata et al. [10,14] and by other authors [3,5] investigating creep behaviour of asphalt mastic characterisation. Although SSCT is more time-consuming, it provides higher consistency and stronger physical relevance for asphalt mastic evaluation.

## 6. Summary and Conclusions

This study investigates the suitability of the Single Shear Creep Test (SSCT) as a robust rheological method for evaluating the high-temperature performance of asphalt mastic in comparison to the commonly used Multiple Stress Creep Recovery Test (MSCRT). The asphalt mastic is defined as a blend of an asphalt binder and mineral filler, where the filler consists of particles smaller than 0.063 mm. This study includes 45 asphalt mastic variants prepared by using 11 different types of asphalt binder, including unmodified, polymer modified, and aged binders, 15 different mineral fillers, and a wide range of different f/b ratios. All variants were tested in a Dynamic Shear Rheometer (DSR) using both the MSCRT and the SSCT at a test temperature of 60 °C. The MSCRT gives empirical parameters like the non-recoverable creep compliance and percent recovery, while the SSCT measures the steady-state creep rate under constant shear stress, providing a more physically meaningful indication of the permanent deformation resistance. The findings from these investigations can be summarized as follows:

- Asphalt binder type has a strong influence on asphalt mastic performance, with polymer-modified and aged binders showing positive influence on asphalt mastic performance at high temperatures.
- The SSCT provides clearer differentiation between mastic variants than MSCRT, especially for soft or unmodified asphalt binders where MSCRT recovery values are often low or misleading.
- Filler properties moderate the influence on asphalt mastic behaviour at high temperature. Spearman correlation analysis revealed that ΔRing and Ball and filler density exhibit the strongest correlation with creep rate and creep compliance.
- Spearman correlations also show that the specific surface area and SiO_2_ content have moderate correlations with percent recovery, indicating that mineralogical composition also contributes to the elastic response. Hydrated lime remains the only filler showing a consistently strong stiffening effect across all parameters.
- The f/b ratio has a strong influence on asphalt mastic performance at high temperatures. As the f/b ratio increases, both creep rate and creep compliance decrease significantly, following a logarithmic trend. This confirms the critical role of the f/b ratio in asphalt mastic behaviour at high temperature.
- Both SSCT and MSCRT tests demonstrated acceptable repeatability and consistency across two different DSR devices (Rheometer A and B). The variation between devices remained within the precision limits defined by EN 16659 [8], confirming that both methods are robust and reproducible across different equipment.
- The SSCT requires more testing time than the MSCRT, but this longer loading period is necessary to allow the mastic to reach a steady state.
- Given the empirical limitations of MSCRT and the robust performance of SSCT across binder types, filler variations, and device platforms, this study recommends the inclusion of SSCT in standardized protocols for asphalt mastic evaluation.
- The SSCT is already included in Annex E of the German technical guideline for asphalt mastic testing in the DSR [15]. However, further validation is still needed, particularly through multi-laboratory studies. In addition, future work should investigate the applicability of SSCT at very high f/b ratios to define the upper f/b limits at which the method can still be reliably performed in the DSR.

## Figures and Tables

**Table 1:** Physical and chemical properties of the fillers used in this study.

Filler Type	Abbreviation	SiO_2_ Content [%]	Particle Density [Mg/m^3^]	Rigden Air Voids Content [Vol.-%]	Δ Ring and Ball [°C]	Specific Surface Area [m^2^/g]
**Limestone (Quarry A)**	Lime-A	11.1	2755	32	18.0	7.144
**Limestone (Quarry B)**	Lime-B	6.9	2778	33	17.0	3.993
**Limestone (Quarry C)**	Lime-C	2.2	2711	31	22.0	1.506
**Limestone (Quarry D)**	Lime-D	9.3	2733	35	19.0	9.870
**Dolomite**	Dol	4.7	2864	34	14.0	3.596
**Greywacke**	Grey	59.8	2753	34	24.0	7.373
**Gabbro**	Gab	48.0	2941	39	27.0	6.315
**Basalt**	Bas	49.8	2903	39	19.5	15.550
**Andesite**	And	57.5	2754	40	22.0	7.111
**Granodiorite**	Grano	68.6	2847	35	19.0	1.731
**Granite**	Granit	69.8	2675	38	24.5	3.785
**Rhyolite**	Rhy	71.1	2744	37	22.0	9.491
**Quartzite**	Quaz	98.6	2658	33	11.0	1.758
**Hydrated Lime**	HL	30.1	2304	58	75.5	14.663
**RAP Filler**	RAP	0.5	2632	47	21.5	6.373

**Table 2:** Spearman correlation coefficients between filler properties and SSCT/MSCRT parameters.

Spearman Correlation	SSCT	MSCRT	
Filler properties	Creep rate	Creep Compliance	Percent Recovery
Density [Mg/m^3^]	0.604	0.679	−0.686
Air voids content by Rigden [%]	−0.326	−0.113	0.113
ΔR&B [°C]	−0.744	−0.366	0.280
Specific surface area [m^2^/g]	−0.150	−0.311	0.411
SiO_2_ content	0.186	0.236	−0.268

## Data Availability

The original contributions presented in this study are included in the article. Further inquiries can be directed to the corresponding author.
